# Flavonoid-Enriched Plant-Extract-Loaded Emulsion: A Novel Phytocosmetic Sunscreen Formulation with Antioxidant Properties

**DOI:** 10.3390/antiox8100443

**Published:** 2019-10-01

**Authors:** Letícia Caramori Cefali, Janaína Artem Ataide, Ana Rita Fernandes, Ilza Maria de Oliveira Sousa, Fernanda Cristina da Silva Gonçalves, Samara Eberlin, José Luis Dávila, Angela Faustino Jozala, Marco Vinicius Chaud, Elena Sanchez-Lopez, Joana Marto, Marcos Akira d’Ávila, Helena Margarida Ribeiro, Mary Ann Foglio, Eliana Barbosa Souto, Priscila Gava Mazzola

**Affiliations:** 1 Institute of Biology, University of Campinas (UNICAMP), R. Monteiro Lobato, 255, Campinas, Sao Paulo 13083-862, Brazil; letisc82@yahoo.com.br; 2 Department of Pharmaceutical Technology, Faculty of Pharmacy, University of Coimbra (UC), Pólo das Ciências da Saúde, Azinhaga de Santa Comba, 3000-548 Coimbra, Portugal; janaina.a.ataide@gmail.com (J.A.A.); anaritavfernandes@gmail.com (A.R.F.); esanchezlopez@ub.edu (E.S.-L.); 3 Faculty of Pharmaceutical Sciences, University of Campinas (UNICAMP), Rua Cândido Portinari, 200, Campinas, Sao Paulo 13083-871, Brazil; maryann.foglio@fcf.unicamp.br; 4 School of Medical Sciences, University of Campinas (UNICAMP), R. Sergio Buarque de Holanda, 250, Campinas, Sao Paulo 13083-859, Brazil; ilzamo.sousa@gmail.com; 5 Kosmoscience Group, Campinas 13041-315, Brazil; fernandagoncalves@kosmoscience.com (F.C.d.S.G.); samara@kosmoscience.com (S.E.); 6 Department of Manufacturing and Materials Engineering, School of Mechanical Engineering, University of Campinas (UNICAMP), Rua Mendeleyev 200, Campinas, São Paulo 13083-860, Brazil; jose.davila@cti.gov.br (J.L.D.); madavila@fem.unicamp.br (M.A.d.); 7 Faculty of Pharmaceutical Sciences, University of Sorocaba (UNISO), Sao Paulo 18023-000, Brazil; angela.jozala@prof.uniso.br (A.F.J.); marco.chaud@prof.uniso.br (M.V.C.); 8 Department of Pharmacy, Pharmaceutical Technology and Physical Chemistry, Faculty of Pharmacy, Institute of Nanoscience and Nanotechnology (IN2UB), University of Barcelona, 08028 Barcelona, Spain; 9 Networking Research Centre of Neurodegenerative Disease (CIBERNED), Instituto de Salud Juan Carlos III, 28031 Madrid, Spain; 10 Research Institute for Medicines (iMed.ULisboa), Faculty of Pharmacy, Universidade de Lisboa, 1649-003 Lisboa, Portugal; jmmarto@ff.ulisboa.pt (J.M.); hribeiro@campus.ul.pt (H.M.R.); 11 Centre of Biological Engineering (CEB), University of Minho, Campus de Gualtar, 4710-057 Braga, Portugal

**Keywords:** flavonoids, phytocosmetic, emulsion, antioxidant, photostability, sunscreen

## Abstract

The aim of this study was to develop a phytocosmetic sunscreen emulsion with antioxidant effect, containing a blend of flavonoid-enriched plant extracts. In vitro sun protection factor, antioxidant activity, skin irritation, photostability, cutaneous permeation, and retention of flavonoids were evaluated. Thermodynamically stable emulsions were obtained and tested for sensorial analysis after loading the blend of extracts. The selected emulsion was stable when stored at low temperatures (5 °C), for which after 120 days the concentration of quercetin and rutin were above their limit of quantification, i.e., 2.8 ± 0.39 μg/mL and 30.39 ± 0.39 μg/mL, respectively. Spreadability, low rupture strength and adhesiveness were shown to be similar to a conventional topical product. Higher brittleness, pseudo-plastic, and viscoelastic behaviors were also recorded for the developed phytocosmetic sunscreen. The product presented a critical wavelength of 387.0 nm and ultraviolet rays A and B (UVA/UVB) rate of 0.78, confirming that the developed formulation shows capacity for UVA/UVB protection, protecting skin against damages caused by ultraviolet (UV) radiation. Rutin was shown to permeate the skin barrier and was also quantified in the stratum corneum (3.27 ± 1.92 μg/mL) by tape stripping and retention test (114.68 ± 8.70 μg/mL). The developed flavonoid-enriched phytocosmetic was shown to be non-irritant to skin by an in vitro assay. Our results confirm the antioxidant activity, sun protection, and physical properties of the developed phytocosmetic for topical application.

## 1. Introduction

Ultraviolet (UV) radiation causes damage to skin, inducing changes in collagen and elastic fibers. The associated photocarcinogenic injuries directly promote DNA damage, which is associated with acceleration of skin aging and risk of skin cancer [1,2]. These risks can be prevented by protecting the skin against exposure to solar radiation, e.g., using sunscreens [3,4]. Sunscreens can be composed of physical and/or chemical filters. Chemical filters are known to cause allergic reactions, contact sensitivity, vitamin D deficiency, photogenotoxicity, among other disorders [5,6,7,8,9]. Potential endocrine disruptors of typical UV filters include benzophenones, camphor derivatives, and cinnamate derivatives [10]. The use of natural ingredients aiming at reducing the skin damage and irritation caused by sunscreens has been increased [11]. Plant materials with ability to absorb or block UV radiation have been extensively studied in the development of sunscreen products against harmful solar radiation [11]. Amongst several examples, phenolic compounds (such as flavonoids) are playing a leading role as they can absorb UV rays, especially UVA and UVB, in wavelengths between 200 nm and 400 nm [12].

Flavonoids are a class of natural products present in fruits, vegetables, and beverages; synthesized by plants; and exhibiting many important effects such as protection against pathogens and UVB radiation [12]. Because of their many biological effects, quercetin and rutin are the most studied flavonoids, especially because of their antioxidant and anticarcinogenic potential [13,14,15].

The development of a suitable formulation requires the confirmation of the physicochemical stability of the product as this is associated to quality control, consumer acceptance, and product efficacy, being an essential test in new cosmetic formulations [16]. Rheological behavior and thermal analysis are also important parameters to be considered during product development, as they allow the follow-up of physicochemical stability of the formulations [17,18].

Cutaneous retention versus permeation of sunscreen components through the skin are also subject of consideration because these products should remain onto the skin surface and no absorption should occur, thereby ensuring product efficacy and safety [19]. Sun filters should not undergo modification when exposed to sunlight, therefore the photostability assay also becomes mandatory. According to Huong et al. [20] and Romanhole et al. [21], the instability of sun filters can decrease their absorptive capacity and consequently their capacity to protect the skin against harmful effects from solar radiation.

The aim of this study was to develop a o/w emulsion phytocosmetic formulation to be used as sunscreen, containing a blend of plant extracts enriched in flavonoids. The sun protection factor, in vitro antioxidant activity, physicochemical stability, photostability, and cutaneous permeation/retention of flavonoids have been determined.

## 2. Materials and Methods

### 2.1. Material

Freeze-dried extracts obtained from *Ginkgo biloba* L., *Dimorphandra mollis* Beth, *Ruta graveolens* and *Vitis vinifera* L. leaves from local Brazilian market were used. A blend of extracts (1:1:1:1) exhibiting in vitro sun protection factor (SPF) values of 8.31 ± 0.5 from *G. biloba* L., 7.72 ± 0.4 from mixed sample, 7.08 ± 0.4 from *R. graveolens* L., 5.04 ± 0.2 from *D. molli* Benth, and 3.71 ± 0.5 from *V. vinifera* L. was used in this work. Emulsions were prepared using tribehenin and a mixture consisting of sorbitan stearate and sucrose cocoate as emulsifiers obtained from Croda (Campinas, São Paulo, Brazil). A mixture of sucrose palmitate glyceryl stearate and sucrose and glyceryl stearate citrate and xanthan gum and manna supplied by Croda (Campinas, São Paulo, Brazil) was used as thickener. Caprylic/capric triglyceride, hydrolyzed wheat protein/polyvinylpyrrolidone (PVP) cross-polymer and *Persea gratissima* (avocado) oil were also supplied by Croda (Campinas, São Paulo, Brazil). Phenoxyethanol, glycerin and talc were provided by PharmaSpecial (São Paulo, Brazil). Isopropanol and ethyl alcohol were provided by Synth (São Paulo, Brazil), 1,1-diphenyl-2-picrylhydrazyl (DPPH), methanol (High Performance Liquid Chromatography (HPLC) grade), formic acid and MTT [3-(4,5-dimethylthiazol-2-yl)-2,5-diphenyltetrazolium bromide, thiazolyl blue tetrazolium bromide] by Sigma-Aldrich (São Paulo, Brazil). Phosphate buffered saline and sodium dodecyl sulphate were provided by Gibco (Waltham, MA, USA). Quercetin (93.3% of purity) and rutin (97.3% of purity) analytical standards by Acros (Itu, Brazil).

### 2.2. Development of o/w Emulsions and Stability Study

Nine (F1 to F9) oil-in-water (o/w) emulsions were produced according to the combinations depicted in Table 1, by heating both phases (aqueous and oily) up to 70 ± 3 °C and then homogenized under cooling until 25 ± 3 °C. Aqueous phase, composed of phenoxiethanol, glycerin, and water, was heated at 70 °C in a beaker using a heating plate (Quimis, São Paulo, Brazil). The oil phase, composed of the remaining components listed in Table 1 (except talc), was heated at the same temperature in a beaker using a heating plate (Quimis, São Paulo, Brazil) until complete melting. The aqueous phase was then poured into the oil phase under manual stirring followed by cooling down to 35 °C. Upon the production of the emulsion, talc was added under manual stirring.

To evaluate the physical stability of emulsions, 5.0 g of each samples were subjected to three centrifugal cycles at 3000 rpm for 30 min in each cycle. This assay was performed at 27 ± 2 °C. Organoleptic characteristics (color, odor, and appearance), pH (Quimis pHmeter, São Paulo, Brazil), density, and viscosity values were also assessed. Density was calculated based on the difference between pycnometers weight (without and with 5 mL of sample) divided by the sample volume [22]. Viscosity assay was carried out at 1.5 rpm rotation for 30 s using spindle number 4 and rotational viscometer (Brookfield, Mod LV-T, São Paulo, Brazil) [16]. The result was expressed in centipoise (cP) and the assay was performed at 27 ± 2 °C.

### 2.3. Sensorial Analysis

Emulsions with better outcomes from the stability tests were subjected to sensorial analysis (number: 59552216.3.0000.5404—approved by the Ethics Committee of University of Campinas), using the sensory difference and preference tests [23]. After signing the informed consent, fifty volunteers (37 women and 13 men) aged between 20 and 50 years old have selected the test emulsions randomly. Volunteers were given 0.1 g of the produced emulsion to be evaluated for the speed absorption, residual fatty sensorial, speed drying, stickiness, spreading, and dry touch, rating each parameter on a scale (like and dislike) [24,25,26]. The best emulsion was selected for the loading of flavonoids.

### 2.4. Production of Phytocosmetic and Stability Study

A phytocosmetic was prepared by adding 200 mg of blend of plant extract (*Ginkgo biloba* L., *Dimorphandra mollis* Beth, *Ruta graveolens* and *Vitis vinifera* L.—1:1:1:1) to the emulsion selected from the sensorial analysis, corresponding to 0.2% of bend extract in the final formulation. The obtained phytocosmetic was then subjected to a stability study [27,28,29]. Stability study was conducted using the multisample analytical centrifuge Lumisizer^®^ (LUM, GmbH, Berlin, Germany). Plain emulsion (without the plant extract) and the developed phytocosmetic (emulsion with the plant extract) were diluted in distilled water (1:5; *w*/*w*) and evaluated for 2 h at 3000 rpm and at 27.5 ± 0.5 °C [30].

The concentration of flavonoids—quercetin and rutin—was determined by High Performance Liquid Chromatography (HPLC) with diode array UV/vis detector. Emulsions submitted to stability study were dissolved in isopropanol (1:10, *w*/*v*) and filtered in 0.45 μm membrane (Merck, Darmstadt, Germany). Quercetin (50 μg/mL) and rutin (500 μg/mL) of analytical standard were analyzed by HPLC for comparison. Samples (5 μL) were injected in HPLC-DAD (Agilent, Technologies 1250 infinity, Santa Clara, United States) with flow rate of 0.3 mL/min for 10 min, at 27 ± 1 °C, using methanol grade HPLC acidified with 0.1% (*v*/*v*) of formic acid (Synth, São Paulo, Brazil) as the mobile phase. Flavonoids identification was carried out at 257 nm in a monomeric chromatographic column C_18_ (Phenomenex, Alcobendas, Spain).

### 2.5. Zeta Potential

Plain emulsion (without the plant extract) and the developed phytocosmetic (emulsion with the plant extract) were diluted in distilled water (1:500; *w*/*w*), and 1 mL of samples was subjected to Zetasizer^®^ equipment (Malvern Instruments Ltd., Malvern, Worcestershire, UK) at 25.0 ± 0.1 °C. Results were obtained of 10 measurements for each sample and assay was carried out in triplicate [31,32].

### 2.6. Droplet Size Distribution

For the determination of the droplet size distribution, each plain emulsion (without the plant extract) and developed phytocosmetic (emulsion with the plant extract) was diluted in distilled water prior to the analysis by laser diffraction using particle size analyzer (MasterSizer^®^, Malvern, UK) and large volume sample dispersion units (Malvern Hydro 2000 MU, Malvern, Germany). Study was carried out at 750 rpm using obscuration range of 10–20% and water as dispersing liquid. Assays were performed in triplicate.

### 2.7. Mechanical Analysis

The textural analysis was performed in a texturometer (Stable Micro Systems TA-XT2i, Godalming, UK), using compression mode. Penetration test was carried out in triplicate at 3 mm/s and applying 0.05 N. A cylindrical probe (SMS P/1R) was used and parameters such as rupture strength (G), adhesiveness (g.sec) and brittleness (mm) were determined. A load cell of 5 kg was used.

Firmness (G) was evaluated using the extrusion cell (HDP/FE) and cylinder probe of 5 mm, measured in triplicate [33,34].

For the spreadability assay, one gram of each sample was introduced in the glass plate, and other glass plate of known weight (420 ± 1 g) was placed over the sample. After 1 min, diameter was read with the aid of millimetric graph paper scale. This procedure was repeated successively by adding other plates of the same weight in one-minute intervals until spreading stopped. All measurements were performed in triplicate, and results were expressed as the spreadability of samples against the applied weight.

The rheological behavior was evaluated using a Rheometer (Anton Paar, MCR 102, Ostfildern, Germany) and a cone-plate sensor (CP50-1). The data were analyzed with Rheoplus V3.61 software. Initially, the flow curve was obtained to determine the hysteresis area through an up-down test. The shear rate was applied from 0 to 100 s^−1^ for the upward curve and from 100 to 0 s^−1^ for the downward curve. The recovery test was then performed in three intervals. In the first interval, a shear rate of 1 s^−1^ was applied for 20 s. Then, in the second interval, a shear rate of 100 s^−1^ was applied for 60 s, and finally, in the third interval, a shear rate of 1 s^−1^ was applied for 100 s to analyze the samples recovery. Then, the amplitude sweep was performed at a constant angular frequency of 10 rad s^−1^ to define the linear viscoelasticity (LVE) range (*γ*_0_ = 0.1% for all samples). Subsequently, the frequency sweep test was performed in a frequency range from 240 to 0.1 rad s^−1^, to evaluate the viscoelastic behavior of the samples. All assays were performed in triplicate at a plate temperature of 25 °C using a solvent trap [35,36,37].

### 2.8. Thermal Analysis

The samples were subjected to differential scanning calorimetry (DSC) and thermogravimetry (TG) analyses, both carried out in triplicate. Approximately 10 mg of sample were placed in an aluminum straw for DSC analysis (Mettler DSC 823e System, Mettler Toledo, Spain), at a heating rate of 15 °C/min in N_2_ atmosphere, from 25 to 350 ± 1 °C temperature. For TG analysis (TA-50WSI, SHIMATZU, 50WSI, Tokyo, Japan), approximately 10 mg of sample were placed in an aluminum straw and analyzed at 10 °C/min heating rate in N_2_ atmosphere with a temperature range of 25 to 500 ± 1 °C [18].

### 2.9. Microbiologic Control

To determine bacteria and fungi growth, one gram of each sample (plain emulsion and phytocosmetic) was diluted in phosphate buffer (pH 7.2; 1:9; *w*/*v*). A volume of 1 mL of the obtained solution was then added to thioglycolate agar (at 35 °C for 24 h) and Sabouraud (at 25 °C for seven days). Bacteria such as *Escherichia coli*, *Staphylococcus aureus*, *Salmonella* sp and *Pseudomonas aeruginosa* were checked using MacConkey agar, bismuth sulfite agar, cetrimide agar and Vogel and Johnson agar as culture media. The dishes were incubated at 35 °C for 24 h and assays were carried out in triplicate [38,39].

### 2.10. In Vitro Sun Protection Factor (SPF) Evaluation of the Phytocosmetic

In Vitro solar protection factor of phytocosmetic was determined by UV spectrophotometry (290 to 320 nm) using the following equation [40]: SPF = CF × $\Sigma_{290}^{320}$ × EE_(λ)_ × I_(λ)_ × Abs_(λ)_, where SPF stands for solar protection factor, CF for correction factor, EE_(λ)_ is the erythemogenic effect of wavelength radiation (λ) nm, and I_(λ)_ is the intensity of solar radiation in the wavelength (λ) nm [41]. The adopted CF (value of 10) and EE_(λ)_ values were previously calculated according to Sayre et al. [42]. Absorbance (Abs_(λ)_) was spectrophotometric read to determine the SPF values. Using spectral transmittance (Labsphere^®^ UV-2000S, Labsphere Halma Company, São Paulo, Brazil) in vitro UVAPF, UVA/UVB rate, and critical wavelength (λc) were also determined as described in the ISO24443:2012 protocol [43]. The measures were carried out in triplicate at 290–400 nm.

### 2.11. In Vitro Antioxidant Activity Analysis of the Phytocosmetic

For the in vitro antioxidant activity, DPPH radical was used. One gram of formulation was diluted in 10 mL of isopropanol. A volume of 2.5 mL of this solution was placed in the test tubes and then 2.5 mL of 0.004% DPPH solution (*w*/*v*) in ethanol was added. The reaction was carried out for 30 min stored under light protection. Analysis was also performed for quercetin as an antioxidant standard at concentrations 0.25, 0.5, 1.0, 1.75, and 2.5 μg/mL. Ethanol was used as blank for maximal absorbance determination at 513 nm. In the presence of DPPH radical scavengers, absorbance intensity decreased, and the percentage of inhibition (% Inhibition) was calculated according to Socorro et al. [44].

### 2.12. Skin Permeation

Two-hundred milligrams of phytocosmetic were placed on synthetic cellulose membranes (Millipore, 0.45 μm) mounted on diffusion cells to determine flavonoids release from emulsion. The assay was carried out in phosphate buffer, at pH 7.2 (68.4 mL of 1M Na_2_HPO_4_ and 31.6 mL of 1 M NaH_2_PO_4_) as receiving medium at 37 °C. To evaluate the flavonoids permeation, cleaned pig skin was placed on diffusion cells with the dermis in contact with the buffer. Rutin concentration was quantified by HPLC/DAD [45], for which the buffer was collected after 1, 2, 4, and 6 h and after 2, 4, 8, 12, and 24 h for release and permeation assays, respectively.

At the end of the tests, skins were washed using distilled water and dried with absorbent paper. Tape-stripping method was performed to determinate rutin concentration on the stratum corneum. Twenty strippings were done using adhesive tape (Dsquame D100, 22 mm—Monaderm, Monaco). Tapes were transferred to tubes with methanol (4 mL) and subjected to shaking for one minute and sonication for 30 min. The supernatant was analyzed by HPLC/DAD.

To evaluate the permeated amount of rutin, after removal of the stratum corneum, skins were cut and transferred to tubes with methanol (4 mL), subjected to shaking for one minute and sonication for 30 min. The supernatant was analyzed by HPLC/DAD. All assays were performed in sextuplicate and using the plain emulsion as blank control [46].

### 2.13. Photostability Study

To evaluate the photostability of phytocosmetic, the samples (phytocosmetic and plain emulsion) were placed on PMMA substrate (HELIOPLATE HD 6–50 mm × 50 mm, 6 microns) and subjected to irradiation at 1.2 J/cm^2^ for 90 min using the solar simulator/sensor of irradiance (CPS+, 1012014/ SunCal BB 300–400 BST Atlas Material Testing Solutions). The photostability was evaluated using a UV transmittance analyzer (Labsphere^®^ UV-2000S, North Sutton, NH, USA). Assay was carried out in quadruplicate. Non-irradiated plates were used as negative control and, as described in the ISO24443:2012 protocol [43], the approved criteria used was ≤17%.

### 2.14. In Vitro Skin Irritation—Reconstructed Human Epidermis

In Vitro skin irritation was performed using Reconstructed Human Epidermis (RhE), composed of human-derived epidermal keratinocytes, according to the Test Guideline number 439 by Organisation for Economic Co-operation and Development (OECD) Guidelines for the Testing of Chemicals [47]. Formulations with extracts were dosed topically in a 3D Reconstructed Human Tissue model, for 42 min. After 42 h post-treatment incubation period, the tissue sample was placed in MTT [3-(4,5-Dimethylthiazol-2-yl)-2,5-diphenyltetrazolium bromide, Thiazolyl blue tetrazolium bromide] (Sigma-Aldrich, São Paulo, Brazil) solution at 1 mg/mL for 3 h. The blue formazan concentration formed in viable cells was measured at 570 nm using spectrophotometer (Versamax, Molecular Devices, São Paulo, Brazil). To evaluate the reliability of the assay, SkinEthic^TM^ RHE (1% Triton X-100) was used. Negative (phosphate buffered saline) (Gibco Waltham, MA, USA) and positive controls (5% aqueous sodium dodecyl sulphate) (Gibco Waltham, MA, USA) were used. According to the literature [47] and as described in the UN GHS (United Nations Globally Harmonized System) Category 2, a sample is considered irritant to skin if the tissue viability is less than or equal to (≤) 50%, after exposure and post-treatment incubation period.

### 2.15. Statistical Analysis

All assays were carried out in three or more replicates, and statistical analysis was carried out using ANOVA test (*p* < 0.05) and Origin (v8.0) software for Windows.

## 3. Results and Discussion

Emulsions containing low concentration of fatty material are considered smoother with minimal oily sensation and are shown to be appropriate for topical application [48,49,50,51]. Bearing that in mind, we have selected low-fatty components, considered by ECOCERT of organic nature and with minimal environmental impact [52]. Formulations were white, creamy, with a pH around 6.6 ± 0.5, and density around 1.0 ± 0.01 g/mL. Depending on the concentration of the selected components, formulations exhibited distinct skin sensation and viscosity.

Emulsion stability was evaluated using an analytical centrifuge Lumisizer^®^ allowing stability parameters, such as sedimentation velocity and shelf life prediction, to be directly calculated [53,54]. When submitted to the stability study, F1, F2, F3, and F4 showed a significant viscosity increase (*p* > 0.05), attributed to the presence of the long-chain fatty acid tribehenin in association with sorbitan stearate and sucrose cocoate. F7 showed phase separation towards error during its preparation. Then, these formulations were discarded for future assays.

Lumisizer^®^ also determines the risk of sedimentation of dispersions [53,54,55]. After assay, formulations F5, F6, F8, and F9 showed signs of sedimentation. However, F5 and F8 were more stable, which may be explained by the presence in F8 of the thickening agent tribehenin and in F5 of higher concentration of polymeric-based oil-in-water emulsifier (sucrose palmitate and glyceryl stearate and glyceryl stearate citrate and sucrose and manna and xanthan gum). F5, F6, F8, and F9 were then used for sensorial analysis.

Effective and discriminative methods are the most used approaches in sensorial analysis in cosmetic industries [24,25,56,57,58], and thus they were used in this study. In effective method, volunteers graded formulations from 0 to 9.

**Figure 1:**
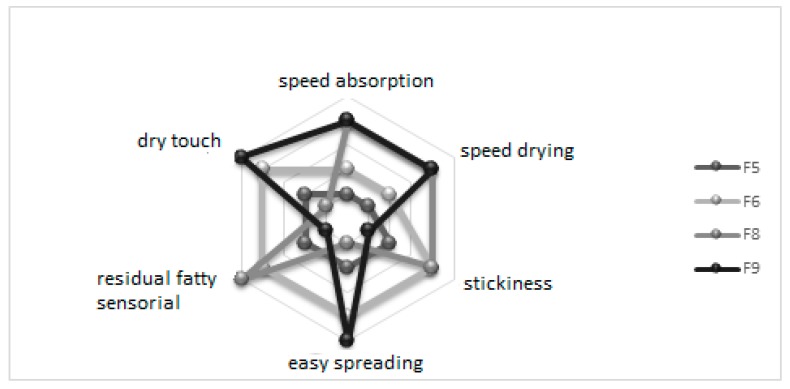
Evaluation of aspects such as speed absorption, residual fatty sensorial, speed drying, stickiness, spreading, and dry touch of base formulations (F5, F6, F8, and F9) by sensorial analysis.

Sample F9 exhibited the best outcome, with 6.8 ± 1.7 grade, followed by F6 (6.7 ± 1.7), F5 (6.1 ± 1.9), and F8 (5.3 ± 2.0). Formulations were evaluated for their sensorial characteristics, and F9 exhibited the best results in absorption speed and drying, easy spreading, low residual fatty sensorial and stickiness, and dry touch. F6 showed similar results as F9, whereas F8 had the worst evaluation (Figure 1).

According to these results, F8 was pointed out as the stickiest of all formulations, attributed to the presence of tribehenin. F5 received median grades from volunteers and therefore was discarded for the next steps. F6 was stickier than F9 because of the higher concentration of sucrose palmitate glyceryl stearate, glyceryl stearate citrate, sucrose, and manna and xanthan gums (5.0%) and lower talc concentration (1.0%). F9 was then chosen to be loaded with flavonoid-enriched plant extracts to develop a phytocosmetic.

Phytocosmetic produced from F9 loading flavonoid-enriched plant extracts was shown to be creamy, with a shiny appearance, yellowish color, and a characteristic odor. The sample presented a pH value compatible with the skin (i.e., 6.80 ± 0.11), density close to 1 g/mL (i.e., 0.96 ± 0.01 g/mL, which translates low amount of air incorporated in the formulation during its preparation), and viscosity of 46,000 ± 1.21 cP. According to the literature, the obtained results are all desirable characteristics for a product to be applied onto the skin [27,28,29,59].

As F9 did not exhibit phase separation after centrifugation, it was then submitted to a stability study. During preliminary assay, the phytocosmetic did not show significant changes in pH, density, and viscosity in all storage conditions (*p* < 0.05). The loading of freeze-dried extracts provided higher stability of flavonoids [60]. Long-term stability of phytocosmetic was evaluated using an analytical centrifuge Lumisizer^®^. Although sedimentation started slightly later in phytocosmetic when compared to the plain emulsion, both presented the same sedimentation behavior and occurred at 115 mm (88%) and at 120 mm (8%). Therefore, blend extract presence did not influence the stability behavior by analytical centrifuge analysis.

Over the period of 90 days, the phytocosmetic darkened when stored in oven (45 ± 2 °C), as expected and previously reported [61,62,63]. Parameters such as pH, density, and viscosity did not show significant changes during storage time (*p* < 0.05).

**Figure 2:**
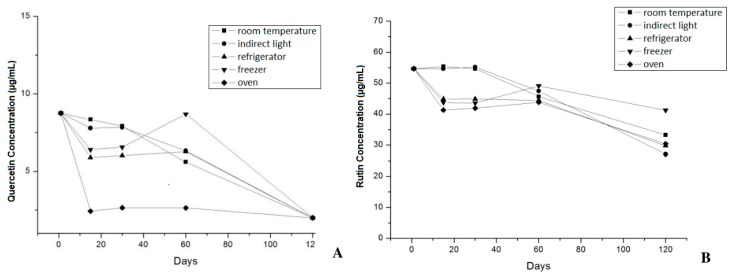
Quercetin concentration (μg/mL) (**A**) and Rutin concentration (μg/mL) (**B**) during stability study of phytocosmetic by HPLC method.

The concentrations of quercetin and rutin determined by HPLC/DAD in the phytocosmetic were 8.75 ± 0.19 μg/mL and 54.67 ± 0.11 μg/mL, respectively. When phytocosmetic was stored in oven, a significant (*p* > 0.05) decrease in flavonoid content was recorded, with quercetin concentration below the quantification limit (2.8 μg/mL) and 30.39 ± 0.39 μg/mL of rutin after 120 days (Figure 2A,B).

In this assay, quercetin concentration increased after 60 days of storage in the freezer. This result is attributed to the higher risk of hydrolysis that rutin shows over storage in comparison to quercetin because of the different glycoside portions of both compounds [64]. Regardless of this trend, concentration of both flavonoids decreased after 120 days of study because of the oxidative process commonly observed in formulations containing vegetable compounds. In addition, it was possible to observe that the phytocosmetic showed higher stability when stored under protection from heat and light.

In this study, plain emulsion and phytocosmetic exhibited zeta potential values of +35.73 ± 1.6 and +30.76 ± 1.9 mV, respectively. According to Jeong et al. [65] and Wiacek and Chibowski [66], a zeta potential of approximately +30 mV is required to prevent flocculation and coalescence, confirming appropriate characteristics of the developed emulsion. The loading of extracts decreased the zeta potential of the formulation, attributed to the presence of solvent residues. This result is in line with Zanatta et al. [67], who also reported differences in the zeta potential values of emulsions with and without plant components.

Laser diffraction method was used to determine droplet size distribution and D_10_, D_50_, and D_90_ values were of 6.84, 23.34, and 57.25 μm, respectively (*p* < 0.05). Furthermore, samples were monodispersed, with a constant profile of droplet size distribution thereby limiting the risk of coalescence. This result suggests enhanced stability of the phytocosmetic, i.e., the loading of plant extract did not induce significant size distribution changes.

Phytocosmetic and plain emulsion were submitted to texture analysis set-up to measure the mechanical properties, as rupture strength, brittleness, adhesiveness, and firmness (Table 2) [68].

The values of rupture strength, brittleness, and firmness recorded for the phytocosmetic and for the base formulation were significantly different (*p* > 0.05). This decrease observed in the values recorded for the phytocosmetic was attributed to the presence of the extract, which contributed to make the product texture lighter. Topical formulations should exhibit acceptable mechanical characteristics, such as easy application and suitable adhesiveness, enabling skin adhesion. In this study, both tested formulations presented desirable parameters for the application of a product onto the skin surface, such as low rupture strength and adhesiveness, and higher brittleness values. Nevertheless, formulations showed high firmness due to the use of polymeric-based oil-in-water emulsifier. This result was similar to that reported by Tai et al. [68] in an anti-inflammatory cream. Although the latter had high firmness, emulsifiers containing polymers can enhance product stability.

Phytocosmetic was subjected to spreadability assay presenting a value of 6079.04 ± 0.44 mm^2^ when subjected to first plate and 13,677.84 ± 0.43 mm^2^ after the sixth plate. Plain emulsion was also evaluated, and no significant difference was observed (*p* < 0.05).

Emulsions are composed by asymmetric particles, which create a non-Newtonian behavior. This latter is required for cosmetic products that suffer deformation when applied onto the skin.

**Figure 3:**
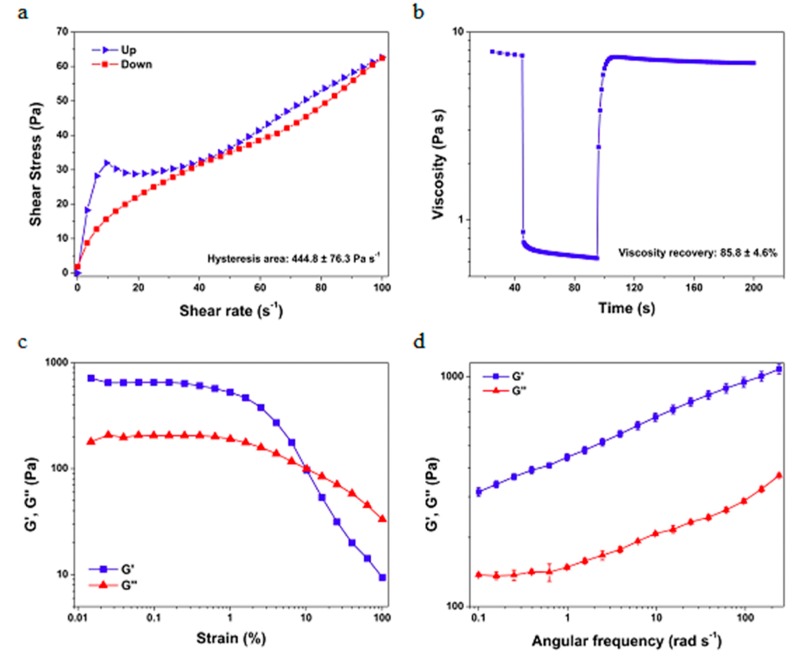
Rheograms of flow curve assay (**a**); fluency and relaxation assay using sheering tension of 7 Pa for 45 s for fluency, 70 Pa for 60 s for resting 7 Pa for 100 s for relaxation (**b**); amplitude and frequency sweep tests (**c**,**d**) of phytocosmetic.

As observed in Figure 3a, a hysteresis area is defined within the upward and the downward flow curves. This area is associated with the energy required to break down the phytocosmetic structure. When the shear application is ceased, thixotropic materials have the ability to rebuild their internal structure to an initial state. In this case, the phytocosmetic exhibits a partial viscosity recovery of approximately 86% after 100 s of the shear cessation, which is illustrated in Figure 3b. The elastic and viscous behaviors of the formulation were assessed by amplitude and frequency sweep tests, which is illustrated in Figure 3c,d.

As observed, the material has a gel-like (G′ > G″) behavior at small strain values. Then, a transition to the liquid-like (G′ < G″) behavior is observed at a strain of approximately 10%, which also guarantees that the material can easily flow on the skin. On the other hand, in the frequency sweep test a gel-like behavior is observed in all the analyzed range, which ensures that the phytocosmetic can maintain its internal structure without deformation into the packing. The elastic behavior predominance is commonly observed in oil-in-water emulsions.

Thermal methods of analysis, such as DSC and TG, were used in this study for determination of the physicochemical stability of emulsions as a function of the temperature variation [69].

According to Table 3, plain emulsion and phytocosmetic presented similar behavior attributed to the presence of endothermic events at approximately 52 °C (stage 1) and 115 °C (stage 2), demonstrating loss of water and volatilization of other emulsions components [70].

Similar results were reported by Schnitzler et al. [71] and Cefali et al. [36] in which o/w emulsions were also subjected to DSC analysis and showed endodermic peaks in the same temperature ranges, demonstrating change of compound’s physical state with temperature variation. Furthermore, according to the obtained data, both formulations showed similar values where endothermic peaks were identified.

Termogravimetry assay exhibited three events that occurred due to formulations’ weight loss (Table 4), showing the loss of free and retained water from sample and of other components around 86 °C (stage 1), 172 °C (stage 2), and 280 °C (stage 3), presenting weight loss around 80%, 4% and 5%, respectively.

Similar results were found by Daneluti et al. [70], in which after thermogravimetric analysis emulsions exhibited a weight loss of 86% and 95%, respectively. In comparison to DSC data, formulations showed similar results in weight loss, while the presence of extracts in the emulsion did not influence the thermal behavior.

Phytocosmetic analysis did not show growth of microorganisms (data not shown). Total count of bacteria, fungi, and yeasts was found lower than 10 CFU in 1.0 g of sample, showing final product quality [38,39].

Formulation containing blend extracts presented a SPF of 2.94 ± 0.4 using Mansur method [40], and of 2.4 ± 0.5 of UVA protection by diffuse reflectance spectroscopy method. The product presented a critical wavelength of 387.0 nm and UVA/UVB rate of 0.78, confirming that the developed formulation shows capacity for UVA/UVB protection, protecting skin against damages caused by UV radiation [29,72]. These in vitro methods are preliminary assays; even though formulation presented FPS value above 2.0 required by the Food and Drug Administration (FDA) in vivo assay [73], interaction between product and skin has not been performed. Besides that, to limit the risk of instability of flavonoids, the concentration of bend extract in the final formulation was low. Formulation containing blend extracts is therefore a promising product to be used as chemical sun filter with UVA/UVB protection that can be associated with physical sun filters increasing the activity. This phenomenon was observed in the study carried out by Seok et al. [74] in which an emulsion containing 24% of physical sun filter (zinc oxide) exhibited a SPF increase from 17.8 to 22.7 when adding 5% of *Scutellaria radix* extract in butanol. Vehicle was evaluated and did not present SPF value by any of the used methods.

Phytocosmetic was subjected to in vitro antioxidant assay against DPPH free radical showing a 17.96 ± 0.03% of inhibition. Other studies using in vivo methods or using other free radicals can be performed to confirm the obtained data. However, this result was similar to that found by Cefali et al. [36], in which cosmetic containing carotenoid exhibited a 19.51% of inhibition. Results of antioxidant activity were lower than single extracts against DPPH (IC_50_ of 174.51 ± 1.1 μg/mL to *D. molli* Benth; 8.12 ± 0.8 μg/mL to *G. biloba* L.; 281.02 ± 1.0 μg/mL to *R. graveolens* L.; 296.90 ± 1.2 μg/mL to *V. vinifera* L., and 28.73 ± 0.7 μg/mL to mix sample) (data not shown). These results indicate a promising antioxidant ability that could aggregate anti-aging property to the formulation, since antioxidants substances can scavenger free radicals from sun, especially the singlet oxygen, decreasing sun damages.

**Figure 4:**
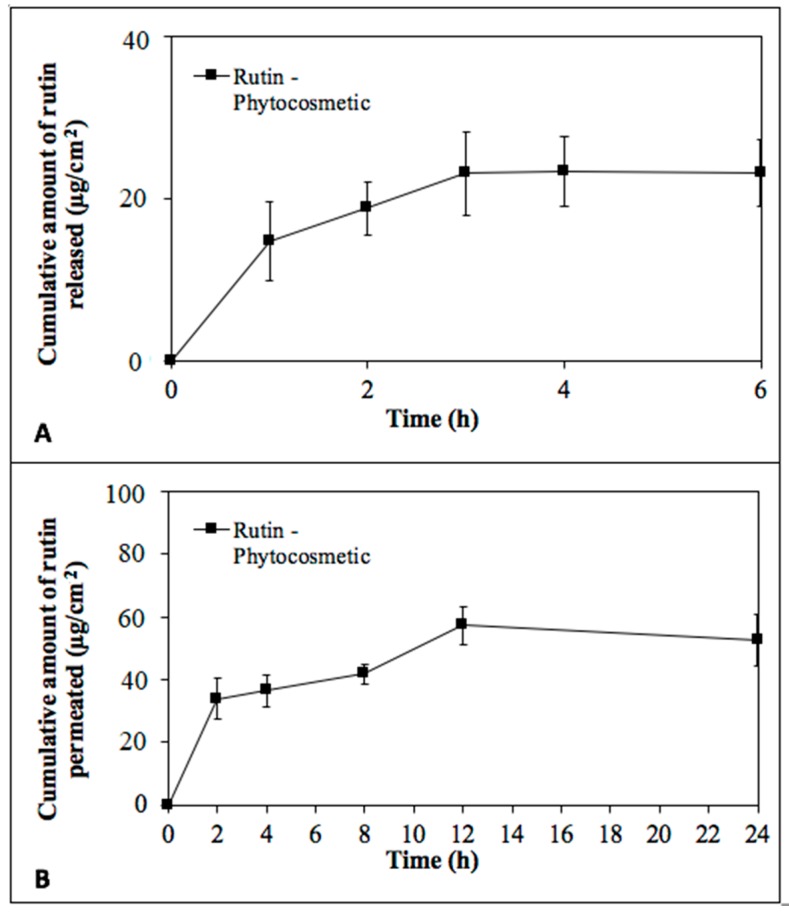
Rutin concentration of μg/mL from phytocosmetic by release test (**A**) and by permeation test (**B**).

Phytocosmetic allowed the release of rutin through a synthetic membrane (after 1, 2, 4, and 6 h) in a receiving medium collected and analyzed using HPLC/DAD (Figure 4A). Rutin was also found in receiving medium in permeation test using pig skin as membrane. This assay was performed for 24 h and rutin was determined in all aliquots obtained during study (Figure 4B) and in ascending amounts until 12 h.

Although rutin permeated the skin, the compound was also found in the stratum corneum (3.27 ± 1.92 μg/mL) by tape stripping test and in higher concentration in the retention test (114.68 ± 8.70 μg/mL). Studies performed by Chuang et al. [75] and Isaac et al. [76] showed release and deposit of flavonoids when incorporated in topical formulations. Moreover, Chuang et al. [75] determined that the viable skin, specially the stratum corneum, could be a barrier delaying flavonoid permeation by enhancing compound deposition. Rutin’s amphiphilic characteristic [64] retained the compound in the skin (stratum corneum and epidermis/dermis) allowing antioxidant activity and sun protection, which can be a promising and desirable specification for anti-aging and sunscreen protection.

Photoproducts generated during absorption of UV radiation process [77,78,79] can cause damages, such as dermatitis and photo-allergies, or be ineffective as sun filters [77,80]. Therefore, photostability evaluation is mandatory for formulations containing new sun filters, ensuring product efficacy and safety [29,81]. Product photostability assay demonstrated a decrease in SPF value down to 2.1 ± 0.4 after 90 min of irradiation (loss of 13.04%). Thus, the product can be considered photostable. According to Hojerova et al. [82], the protocol considers that a formulation can lose up to 20% of the SPF after being subjected to artificial UV radiation. That was an expected result for the formula containing antioxidant materials such as flavonoids.

Studies performed by Jarzycka et al. [83] and Choquenet et al. [84] found similar results, reporting that quercetin is capable to reduce the photodegradation of chemical sun filters without any changes in the effectiveness of the formulation [85,86].

In Vitro skin irritation test describes an in vitro procedure that may be used for the hazard identification of irritant products, as described by the UN Globally Harmonized System of Classification and Labelling (GHS) Category 2. According Kandarova et al. [87], a modified reconstructed human epidermis skin irritation test has the potential to address the skin irritation potential of medical devices. Therefore, in this study, formulation with extracts has reduced reconstructed human epidermis cell viability to less than 50% (Table 5), whereas formulation was shown to be non-irritant by in vitro assay.

These results were according to cell viability assay performed for the extracts, in which all extracts isolated and mixed (200 μg/mL) reduced HaCat cell viability to less than 50% by in vitro assay (data not shown).

## 4. Conclusions

Our flavonoid-enriched emulsion (phytocosmetic) exhibited stability when stored at low temperatures. Properties, such as high spreadibility, low rupture strength and adhesiveness and higher brittleness, shear shinning and viscoelastic behaviors, were suitable for topical application. It exhibited antioxidant activity and UVA protection in in vitro assays, as well as photostability. Phytocosmetic was not irritant to skin, and rutin could be found both in stratum corneum and in deeper epidermis, enhancing antioxidant activity and sun protection effect, both desirable for anti-aging and sunscreen actions. Despite the low SPF value, the developed product is promising to be used as sunscreen, especially if associated with physical sun filters, which would increase its solar protection.

## Figures and Tables

**Table 1:** Components of oil-in-water emulsions.

Components (INCI NAME)	Concentration (%)								
	F1	F2	F3	F4	F5	F6	F7	F8	F9
Tribehenin	5.0	6.0	5.0	6.0	-	-	8.0	8.0	-
Sorbitan stearate and sucrose cocoate	8.0	8.0	8.0	8.0	8.0	8.0	-	-	8.0
Sucrose palmitate glyceryl stearate and glyceryl stearate citrate and sucrose and manna and xanthan gum	5.0	5.0	3.0	3.0	5.0	2.0	5.0	2.0	0.5
Caprylic and capric triglyceride	2.0	2.0	2.0	2.0	2.0	2.0	2.0	2.0	2.0
Hydrolyzed wheat protein and PVP cross-polymer	3.0	3.0	5.0	5.0	3.0	2.0	3.0	2.0	2.0
*Persea gratissima* (Avocado) oil	0.5	0.5	0.5	0.5	0.5	0.5	0.5	0.5	0.5
Phenoxyethanol	0.5	0.5	0.5	0.5	0.5	0.5	0.5	0.5	0.5
Glycerin	3.0	3.0	3.0	3.0	3.0	3.0	3.0	3.0	3.0
Talc	1.0	1.0	1.0	1.0	1.0	1.0	1.5	1.5	1.5
Aqua	72.0	71.0	72.0	71.0	77.0	81.0	76.5	80.5	82.0

**Table 2:** Texture analysis of plain emulsion (without plant extract) and phytocosmetic (emulsion with the blend of plant extract).

Formulations	Rupture Strength (G)	Brittleness (mm)	Adhesiveness (g.s)	Firmness (G)
Plain emulsion	3.62 ± 1.31	19.05 ± 0.76	−50.18 ± 0.63	633.26 ± 0.11
Phytocosmetic	3.07 ± 0.33	16.57 ± 2.90	−43.01 ± 2.53	487.08 ± 0.15

**Table 3:** Temperature values (°C) presented during enthalpy variation of components from plain emulsion and phytocosmetic.

Formulations	Enthalpy Variation	
	Stage 1 (°C)	Stage 2 (°C)
Plain emulsion	51.66	120.93
Phytocosmetic	52.65	113.77

**Table 4:** Percentage of weight loss of components from vehicle and phytocosmetic during the three stages presented by medium temperatures (T_M_) determined.

Formulation	T_M_ (°C) Stage 1	Weight Loss (%)	T_M_ (°C) Stage 2	Weight Loss (%)	T_M_ (°C) Stage 3	Weight Loss (%)
Plain emulsion	87.89	86.64	167.83	3.94	278.22	4.21
Phytocosmetic	82.76	79.87	166.97	4.53	273.20	5.82

**Table 5:** Cell viability percentage of formulation with extracts by in vitro skin irritation assay.

Samples	Cell Viability (%) ± SD
Formulation with extracts	104.6 ± 7.5
Negative control	100.0 ± 2.9
Positive control	1.1 ± 0.1

## References

[1] Baron E.D., Kirkland E.B., Domingo D. (2008). Advances in photoprotection. Dermatol. Nurs..

[2] Palm M.D., O’Donoghue M.N. (2007). Update on photoprotection. Dermatol. Ther..

[3] Arts I.C., Hollman P.C. (2005). Polyphenols and disease risk in epidemiologic studies. Am. J. Clin. Nutr..

[4] Romanhole R.C., Ataide J.A., Moriel P., Mazzola P.G. (2015). Update on ultraviolet A and B radiation generated by the sun and artificial lamps and their effects on skin. Int. J. Cosmet. Sci..

[5] Amar S.K., Goyal S., Dubey D., Srivastav A.K., Chopra D., Singh J., Shankar J., Chaturvedi R.K., Ray R.S. (2015). Benzophenone 1 induced photogenotoxicity and apoptosis via release of cytochrome c and Smac/DIABLO at environmental UV radiation. Toxicol. Lett..

[6] Gilbert L., Picard C., Savary G., Grisel M. (2013). Rheological and textural characterization of cosmetic emulsions containing natural and synthetic polymers: Relationships between both data. Colloids Surf. A Physicochem. Eng. Asp..

[7] Ramos S., Homem V., Alves A., Santos L. (2016). A review of organic UV-filters in wastewater treatment plants. Environ. Int..

[8] Sambandan D.R., Ratner D. (2011). Sunscreens: An overview and update. J. Am. Acad. Dermatol..

[9] Wong T., Orton D. (2011). Sunscreen allergy and its investigation. Clin. Dermatol..

[10] Ruszkiewicz J.A., Pinkas A., Ferrer B., Peres T.V., Tsatsakis A., Aschner M. (2017). Neurotoxic effect of active ingredients in sunscreen products, a contemporary review. Toxicol. Rep..

[11] Cefali L., Ataide J., Moriel P., Foglio M., Mazzola P. (2016). Plant-based active photoprotectants for sunscreens. Int. J. Cosmet. Sci..

[12] De Cooman L., Everaert E., de Keukeleire D. (1998). Quantitative analysis of hop acids, essential oils and flavonoids as a clue to the identification of hop varieties. Phytochem. Anal..

[13] Tohge T., de Souza L.P., Fernie A.R. (2017). Current understanding of the pathways of flavonoid biosynthesis in model and crop plants. J. Exp. Bot..

[14] AMacedo S., Quelhas S., Silva A.M., Souto E.B. (2014). Nanoemulsions for delivery of flavonoids: Formulation and in vitro release of rutin as model drug. Pharm. Dev. Technol..

[15] Santos I.S., Ponte B.M., Boonme P., Silva A.M., Souto E.B. (2013). Nanoencapsulation of polyphenols for protective effect against colon-rectal cancer. Biotechnol. Adv..

[16] Corrêa N.M., Júnior F.B.C., Ignácio R.F., Leonardi G.R. (2005). Avaliação do comportamento reológico de diferentes géis hidrofílicos. Rev. Bras. Ciênc. Farm..

[17] Barry B.W. (1993). Rheology of Dermatological Vehicles.

[18] Ribeiro H., Morais J., Eccleston G. (2004). Structure and rheology of semisolid o/w creams containing cetyl alcohol/non-ionic surfactant mixed emulsifier and different polymers. Int. J. Cosmet. Sci..

[19] Montenegro L., Puglisi G. (2013). Evaluation of sunscreen safety by in vitro skin permeation studies: Effects of vehicle composition. Die Pharm. Int. J. Pharm. Sci..

[20] Huong S.P., Rocher E., Fourneron J.-D., Charles L., Monnier V., Bun H., Andrieu V. (2008). Photoreactivity of the sunscreen butylmethoxydibenzoylmethane (DBM) under various experimental conditions. J. Photochem. Photobiol. A Chem..

[21] Romanhole R.C., Ataide J.A., Cefali L.C., Moriel P., Mazzola P.G. (2016). Photostability study of commercial sunscreens submitted to artificial UV irradiation and/or fluorescent radiation. J. Photochem. Photobiol. B Biol..

[22] Idson B. (1993). Stability Testing of Emulsions-Part I. Drug Cosmet. Ind..

[23] MacFie H.J.H., Thomson D.M.H., Piggott J.R (1988). Preference mapping and multidimensional scaling. Sensory Analysis of Foods.

[24] Almeida I., Gaio A., Bahia M. (2008). Hedonic and descriptive skinfeel analysis of two oleogels: Comparison with other topical formulations. J. Sens. Stud..

[25] Isaac V.L.B., Chiari B.G., Magnani C., Corrêa M.A. (2013). Análise sensorial como ferramenta útil no desenvolvimento de cosméticos. Rev. Ciênc. Farm. Básica Apl..

[26] Dutcosky S.D. (2007). Análise Sensorial de Alimentos.

[27] EMEA (2008). Guideline on Stability Testing: Stability Testing of New Drug Substances and Products.

[28] ISO (2013). Guidelines for the Characterization of Dispersion Stability.

[29] Colipa Project Team IV (2006). Method for the In Vitro Determination of UVA Protection Provided by Sunscreen Products.

[30] Badolato G., Aguilar F., Schuchmann H., Sobisch T., Lerche D. (2008). Evaluation of long term stability of model emulsions by multisample analytical centrifugation. Surface and Interfacial Forces–From Fundamentals to Applications.

[31] Roland I., Piel G., Delattre L., Evrard B. (2003). Systematic characterization of oil-in-water emulsions for formulation design. Int. J. Pharm..

[32] Hejjaji E.M., Smith A.M., Morris G.A. (2017). Designing chitosan-tripolyphosphate microparticles with desired size for specific pharmaceutical or forensic applications. Int. J. Biol. Macromol..

[33] Savary G., Grisel M., Picard C. (2013). Impact of emollients on the spreading properties of cosmetic products: A combined sensory and instrumental characterization. Colloids Surf. B Biointerfaces.

[34] Estanqueiro M., Conceição J., Amaral M.H., Santos D., Silva J.B., Lobo J.M.S. (2014). Characterization and stability studies of emulsion systems containing pumice. Braz. J. Pharm. Sci..

[35] Isaac V.L.B., Cefali L., Chiari B., Almeida M., Ribeiro H., Correa M.A. (2013). Effect of various thickening agents on the rheological properties of oil-in-water emulsions containing nonionic emulsifier. J. Dispers. Sci. Technol..

[36] Cefali L.C., Souza-Moreira T.M., Corrêa M.A., Salgado H.R.N., Isaac V.L.B. (2015). Development and evaluation of an emulsion containing lycopene for combating acceleration of skin aging. Braz. J. Pharm. Sci..

[37] Dávila J.L., d’Ávila M.A. (2017). Laponite as a rheology modifier of alginate solutions: Physical gelation and aging evolution. Carbohydr. Polym..

[38] ISO (2014). Cosmetics—Microbiology—Microbiological Limits.

[39] USP (2013). The United States Pharmacopeial Convention.

[40] Mansur M.C.P., Leitão S.G., Cerqueira-Coutinho C., Vermelho A.B., Silva R.S., Presgrave O.A., Leitão Á.A., Leitão G.G., Ricci-Júnior E., Santos E.P. (2016). In Vitro and in vivo evaluation of efficacy and safety of photoprotective formulations containing antioxidant extracts. Rev. Bras. Farm..

[41] Severino P., Moraes L.F., Zanchetta B., Souto E.B., Santana M.H. (2012). Elastic liposomes containing benzophenone-3 for sun protection factor enhancement. Pharm. Dev. Technol..

[42] Sayre R.M., Agin P.P., LeVee G.J., Marlowe E. (1979). A comparison of in vivo and in vitro testing of sunscreening formulas. Photochem. Photobiol..

[43] ISO (2012). Determination of Sunscreen UVA Photoprotection In Vitro.

[44] Socorro M.R.M.d., Alves R.E., de Brito E.S., Pérez-Jiménez J., Saura-Calixto F., Mancini-Filho J. (2010). Bioactive compounds and antioxidant capacities of 18 non-traditional tropical fruits from Brazil. Food Chem..

[45] Seal T. (2016). Quantitative HPLC analysis of phenolic acids, flavonoids and ascorbic acid in four different solvent extracts of two wild edible leaves, Sonchus arvensis and Oenanthe linearis of North-Eastern region in India. J. Appl. Pharm. Sci..

[46] JAlencastre B., Bentley M.V.L.B., Garcia F.S., Moragas M.d., Viladot J.L., Marchetti J.M. (2006). A study of the characteristics and in vitro permeation properties of CMC/chitosan microparticles as a skin delivery system for vitamin E. Revista Brasileira de Ciências Farmacêuticas.

[47] OECD Test Guideline No. 439. In Vitro Skin Irritation: Reconstructed Human Epidermis Test Method. https://www.oecd-ilibrary.org/docserver/9789264242845-en.pdf?expires=1570612196&id=id&accname=guest&checksum=7598839FA6F86688026A68D7259A6694.

[48] Pimentel-Moral S., Teixeira M.C., Fernandes A.R., Arraez-Roman D., Martinez-Ferez A., Segura-Carretero A., Souto E.B. (2018). Lipid nanocarriers for the loading of polyphenols—A comprehensive review. Adv. Colloid Interface Sci..

[49] Clares B., Calpena A.C., Parra A., Abrego G., Alvarado H., Fangueiro J.F., Souto E.B. (2014). Nanoemulsions (NEs), liposomes (LPs) and solid lipid nanoparticles (SLNs) for retinyl palmitate: Effect on skin permeation. Int. J. Pharm..

[50] Teixeira M.C., Severino P., Andreani T., Boonme P., Santini A., Silva A.M., Souto E.B. (2017). d-alpha-tocopherol nanoemulsions: Size properties, rheological behavior, surface tension, osmolarity and cytotoxicity. Saudi Pharm. J..

[51] Severino P., Fangueiro J.F., Ferreira S.V., Basso R., Chaud M.V., Santana M.H., Rosmaninho A., Souto E.B. (2013). Nanoemulsions and nanoparticles for non-melanoma skin cancer: Effects of lipid materials. Clin. Transl. Oncol..

[52] ECOCERT Organismo de Inspeção e Certificação. Ecocert Brasil. http://www.brazil.ecocert.com/politicas-e-diretrizes-ecocert.

[53] Carbone C., Teixeira M.D.C., Sousa M.D.C., Martins-Gomes C., Silva A.M., Souto E.M.B., Musumeci T. (2019). Clotrimazole-Loaded Mediterranean Essential Oils NLC: A Synergic Treatment of Candida Skin Infections. Pharmaceutics.

[54] Zielinska A., Martins-Gomes C., Ferreira N.R., Silva A.M., Nowak I., Souto E.B. (2018). Anti-inflammatory and anti-cancer activity of citral: Optimization of citral-loaded solid lipid nanoparticles (SLN) using experimental factorial design and LUMiSizer(R). Int. J. Pharm..

[55] Shimojo A.A.M., Fernandes A.R.V., Ferreira N.R.E., Sanchez-Lopez E., Santana M.H.A., Souto E.B. (2019). Evaluation of the Influence of Process Parameters on the Properties of Resveratrol-Loaded NLC Using 2(2) Full Factorial Design. Antioxidants.

[56] Meilgaard M.C., Carr B.T., V G. (1999). Civille, Sensory Evaluation Techniques.

[57] Muñoz A.M., Civille V.G., Carr T.B. (1993). Sensory Evaluation in Quality Control.

[58] Silva T.M.D., Migliato K.F., Salgado H.R.N., Rangel V.L.B.I. (2004). Quality comparison of W/O and O/W photo-protection creams. Cosmet. Toilet..

[59] Isaac V.L.B., Cefali L.C., Chiari B.G., Oliveira C.C.L.G., Salgado H.R.N., Correa M.A. (2008). Protocolo para ensaios físico-químicos de estabilidade de fitocosméticos. Revista de Ciências Farmacêuticas Básica e Aplicada.

[60] Liapis A.I., Bruttini R., Mujumdar A. (2006). Freeze drying. Handbook of Industrial Drying.

[61] Figueiredo S.A., Vilela F.M.P., da Silva C.A., Cunha T.M., Santos M.H.D., Fonseca M.J.V. (2014). In Vitro and in vivo photoprotective/photochemopreventive potential of *Garcinia brasiliensis* epicarp extract. J. Photochem. Photobiol. B Biol..

[62] Hubinger S.Z., Cefali L.C., Vellosa J.C., Salgado H.R.N., Isaac V.L.B., Moreira R.R.D. (2010). *Dimorphandra mollis*: An alternative as a source of flavonoids with antioxidant action. Lat. Am. J. Pharm..

[63] Veberic R., Jakopic J., Stampar F., Schmitzer V. (2009). European elderberry (*Sambucus nigra* L.) rich in sugars, organic acids, anthocyanins and selected polyphenols. Food Chem..

[64] Grinberg L.N., Rachmilewitz E.A., Newmark H. (1994). Protective effects of rutin against hemoglobin oxidation. Biochem. Pharmacol..

[65] Jeong Y.I., Cho C.S., Kim S.H., Ko K.S., Kim S.I., Shim Y.H., Nah J.W. (2001). Preparation of poly (DL-lactide-co-glycolide) nanoparticles without surfactant. J. Appl. Polym. Sci..

[66] Wiącek A.E., Chibowski E. (2002). Zeta potential and droplet size of n-tetradecane/ethanol (protein) emulsions. Colloids Surf. B Biointerfaces.

[67] Zanatta C., Arnal M.M., Urgatondo V., Rocha-Filho P., Martínez-Hidalgo M.V. (2010). Photoprotective potential of emulsions formulated with Buriti oil (*Mauritia flexuosa*) and Vitamin E against UV irradiation on human keratinocytes and fibroblasts cell lines. Food Chem. Toxicol..

[68] Tai A., Bianchini R., Jachowicz J. (2014). Texture analysis of cosmetic/pharmaceutical raw materials and formulations. Int. J. Cosmet. Sci..

[69] Casimiro M.H., Leal J.P., Gil M.H., Castro C. (2005). Análise calorimétrica aplicada a polímeros biológicos. Parte I: Fundamentos teóricos. Boletim da Sociedade Portuguesa de Química.

[70] Daneluti A.L.M., Velasco M.V.R., Baby A.R., Matos J.d.R. (2015). Thermal behavior and free-radical-scavenging activity of phytic acid alone and incorporated in cosmetic emulsions. Cosmetics.

[71] Schnitzler E., Carvalho M.A.D.S.F., Stadler C.C., Volpato A.M., Ionashiro M. (2001). Aplicação da calorimetria exploratória diferencial (dsc) na caracterização térmica do acetato de dexametazona, excipientes e do creme de dexametazona. Eclética Química.

[72] De Flora S., Ferguson L.R. (2005). Overview of mechanisms of cancer chemopreventive agents. Mutat. Res. Fundam. Mol. Mech. Mutagen..

[73] Food and Drug Administration (2019). Sunscreen Drug Products for Over-the-Counter Human Use.

[74] Seok H.K., Kwak J.Y., Choi G.W., An S.M., Kwak J.H., Seo H.H., Suh H.J., Boo Y.C. (2016). Scutellaria radix Extract as a Natural UV Protectant for Human Skin. Phytother. Res..

[75] Chuang S.-Y., Lin Y.-K., Lin C.-F., Wang P.-W., Chen E.-L., Fang J.-Y. (2017). Elucidating the skin delivery of aglycone and glycoside flavonoids: How the structures affect cutaneous absorption. Nutrients.

[76] Isaac V.L.B., Chiari B.G., Miglioli K., Moreira R., Oliveira J.R.S., Salgado H., Relkin P., Correa M.A., Salgado A., Ribeiro H.M. (2012). Development of a topical formulation containing *S. lutea* extract: Stability, in vitro studies and cutaneous permeation. J. Appl. Pharm. Sci..

[77] Gaspar L., Campos P.M. (2003). Rheological behavior and the SPF of sunscreens. Int. J. Pharm..

[78] Mayer L., Wadsley J., Quinn T., Stadel J. (2005). Gravitational instability in binary protoplanetary discs: New constraints on giant planet formation. Mon. Not. R. Astron. Soc..

[79] Perugini D., Poli G., Gatta G. (2002). Analysis and simulation of magma mixing processes in 3D. Lithos.

[80] Antoniou C., Kosmadaki M.G., Stratigos A.J., Katsambas A.D. (2008). Sunscreens—What’s important to know. J. Eur. Acad. Dermatol. Venereol..

[81] Herzog B., Wehrle M., Quass K. (2009). Photostability of UV absorber systems in sunscreens. Photochem. Photobiol..

[82] Hojerová J., Medovcíková A., Mikula M. (2011). Photoprotective efficacy and photostability of fifteen sunscreen products having the same label SPF subjected to natural sunlight. Int. J. Pharm..

[83] Jarzycka A., Lewińska A., Gancarz R., Wilk K.A. (2013). Assessment of extracts of *Helichrysum arenarium*, *Crataegus monogyna*, *Sambucus nigra* in photoprotective UVA and UVB; photostability in cosmetic emulsions. J. Photochem. Photobiol. B Biol..

[84] Choquenet B., Couteau C., Paparis E., Coiffard L.J. (2008). Quercetin and rutin as potential sunscreen agents: Determination of efficacy by an in vitro method. J. Nat. Prod..

[85] De Alencar Filho J.E.M.T., Sampaio P.A., Pereira E.C.V., de Oliveira Junior R.G., Silva F.I.S., da Silva Almeida J.R.G., Rolim L.A.U., Nunes X.P., da Cruz Araujo E.C. (2016). Flavonoids as photoprotective agents: A systematic review. J. Med. Plants Res..

[86] Scalia S., Mezzena M. (2010). Photostabilization effect of quercetin on the UV filter combination, butyl methoxydibenzoylmethane–octyl methoxycinnamate. Photochem. Photobiol..

[87] Kandarova H., Willoughby J.A., de Jong W.H., Letasiova S., Milasova T., Bachelor M.A., Breyfogle B., Handa Y., de la Fonteyne L., Coleman K.P. (2018). Pre-validation of an in vitro skin irritation test for medical devices using the reconstructed human tissue model EpiDerm™. Toxicol. Vitr..

